# Crack Extension and Possibility of Debonding in Encapsulation-Based Self-Healing Materials

**DOI:** 10.3390/ma10060589

**Published:** 2017-05-27

**Authors:** Wenting Li, Zhengwu Jiang, Zhenghong Yang

**Affiliations:** Key Laboratory of Advanced Civil Engineering Materials, Tongji University, Ministry of Education, Shanghai 201804, China; lwt@tongji.edu.cn (W.L.); yzh@tongji.edu.cn (Z.Y.)

**Keywords:** encapsulation-based, self-healing, interface fracture, cohesive zone

## Abstract

The breakage of capsules upon crack propagation is crucial for achieving crack healing in encapsulation-based self-healing materials. A mesomechanical model was developed in this study to simulate the process of crack propagation in a matrix and the potential of debonding. The model used the extended finite element method (XFEM) combined with a cohesive zone model (CZM) in a two-dimensional (2D) configuration. The configuration consisted of an infinite matrix with an embedded crack and a capsule nearby, all subjected to a uniaxial remote tensile load. A parametric study was performed to investigate the effect of geometry, elastic parameters and fracture properties on the fracture response of the system. The results indicated that the effect of the capsule wall on the fracture behavior of the matrix is insignificant for *t_c_*/*R_c_* ≤ 0.05. The matrix strength influenced the ultimate crack length, while the Young’s modulus ratio *E_c_*/*E_m_* only affected the rate of crack propagation. The potential for capsule breakage or debonding was dependent on the comparative strength between capsule and interface (S_c_/S_int_), provided the crack could reach the capsule. The critical value of S_c,cr_/S_int,cr_ was obtained using this model for materials design.

## 1. Introduction

As an alternative to conventional passive repair, bio-inspired self-healing materials are of great interest to material scientists because they have the built-in capability to repair structural damage either autogenously or with minimal help from an external stimulus.

A variety of innovative strategies have been explored and conducted to attempt to endow materials with self-healing abilities [1,2,3,4,5,6,7,8,9,10,11,12,13,14,15,16,17,18,19,20]. Encapsulation-based self-healing materials are one of the most promising techniques in this area, and they are receiving considerable attention since being initially demonstrated in the pioneering work of White et al. [6]. This process essentially involves crack propagation rupturing the embedded microcapsules, and the incorporated healing agent released into the crack face through certain physical mechanisms such as capillary action [6]. The polymerization of the healing agent, which can be triggered by the embedded catalyst, can narrow and even close the detached crack surfaces. 

Therefore, the breakage of capsules takes a primary role in the realization of crack healing. In other words, the capsules need to be prudently designed with the appropriate geometry and mechanical properties for the capsules to break when a crack reaches them. To achieve this, the stress transferred from a crack to the capsules should be high enough to rupture the capsules. Unfortunately, a crack may propagate along the capsule–matrix interface and result in premature debonding at a weak interface. This is particularly problematic for inorganic hosts such as cement-based materials considering encapsulation techniques mostly apply to polymers. In contrast, a polymer matrix is normally chemically compatible with the capsule materials.

The full process of capsule breakage consists of two steps: (i) crack propagation in a matrix prior to reaching a capsule; and (ii) capsule rupture or debonding, depending on the interface properties. The former problem (i) of crack propagation in either homogeneous or heterogeneous solids has been widely investigated and described thoroughly in the literature. Likewise, the effects of an arbitrarily shaped hole near a crack tip on the stress field and stress intensity factor have been analyzed numerically, although this configuration is not a precise representation of the presence of a capsule [21,22,23,24]. A more accurate configuration for this case was proposed in which the stress concentration in a solid with an embedded capsule was evaluated for various interfacial conditions [25]. The onset of an interfacial crack due to a lack of debonding was clearly understood, although no initiation of the crack in the host was included in the study. In a similar manner, the stress concentration has been numerically analyzed but with an additional crack placed in the matrix with its tip right below the capsule [26]. Although this problem is partially understood for some specific cases, these findings provide good insight into further and comprehensive understanding about the complete event of crack propagation in a matrix and the potential of debonding as a result.

In this study, both the crack propagation in a matrix and the capsule–matrix interaction were investigated numerically using the extended finite element method (XFEM) combined with a cohesive zone model (CZM). The combined methods of XFEM and CZM were applied to fracture simulations of asphalt mixtures with heterogeneous microstructures in which a viscoelastic constitutive model was adopted for cracking in a matrix [27]. By taking advantage of XFEM, which does not require mesh to conform to geometric discontinuities, mesh refinement was not required in the region around the crack tip to capture the singular asymptotic fields adequately. The crack propagation in the matrix was exhaustively modeled by changing the mechanics of the matrix. However, it is difficult to simulate the interaction between the crack in a host and the capsule–matrix interface using only XFEM. Therefore, to determine the effect of an interfacial material on potential debonding, the cohesive zone model, achieved by pre-inserted zero-thickness cohesive elements (CIEs), was used to understand the stress transfer between matrix and capsule. It is noteworthy that the configuration proposed in this study also resembles the debonding of cylindrical capsules in a 2D domain. In that sense, this analysis also applies to material design for that case. 

## 2. Numerical Setup

**Figure 1:**
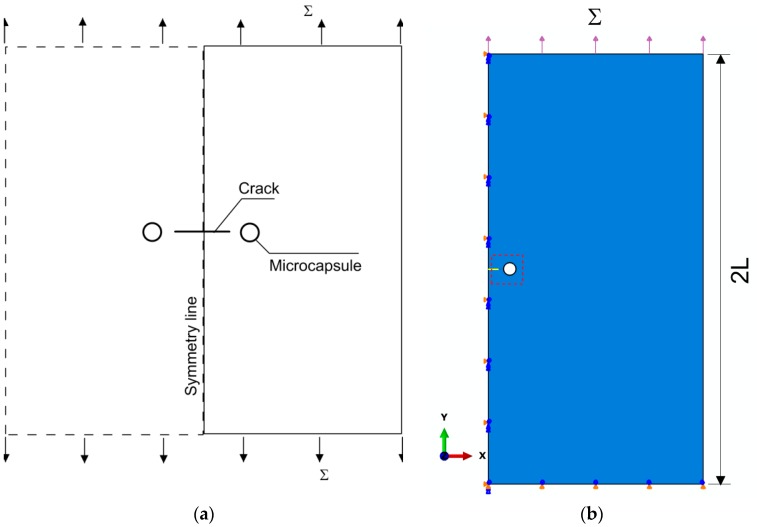
(**a**) Model setup; and (**b**) detailed boundary and loading conditions of symmetrical configuration.

Consider a two-dimensional, infinite, isotropic, and homogeneous geometry of length 2*L* containing two circular holes of outer radius $R_{c}$ and a crack of 2$l_{0}$ in length with its center at the origin in between the two holes, as depicted in Figure 1a. A capsule with wall thickness $t_{c}$ should be inserted and bonded to each hole. This layout should represent the potential for the crack to freely propagate through the capsule on either side. Furthermore, it is assumed that the capsule size is small enough, when compared to the entire domain, to resemble the hypothetical condition of a holed plate with an embedded crack under a remote tensile stress ∑; thus, the edges of the linear crack 2$l_{0}$ are free from traction. Taking advantage of the symmetrical configuration, this analysis has been performed on half of the geometry, as shown in Figure 1b, in which the selected geometry and boundary conditions are schematically illustrated. Specifically, the full length of the domain was set to 2*L* = 10 mm and the outer radius to $R_{c}=$ 150 mm. Three various wall thicknesses ranging in different scales of magnitude were evaluated for $t_{c}/R_{c}$: 0.006, 0.05 and 0.12, which correspond with the true dimensions of microencapsulation-based self-healing specimens in lab tests [28,29,30].

**Figure 2:**
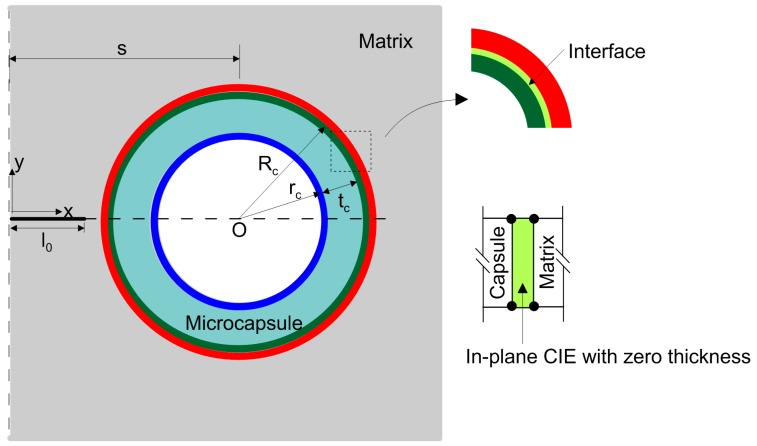
Geometries, surface regions and inserted cohesive elements of the interface.

**Figure 3:**
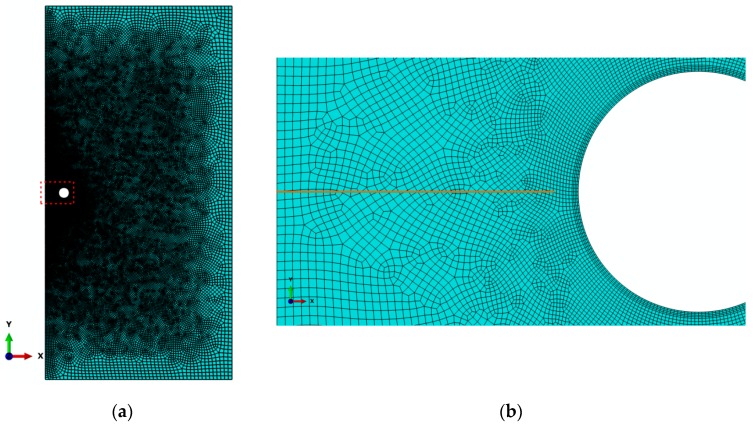
Mesh grid used in this work: (**a**) full mesh of the half model; and (**b**) zoomed view of the vicinity of crack and capsule.

A magnified view of the vicinity around the capsule, as highlighted in the red dashed box in Figure 1b, is schematically shown in Figure 2. The detailed composition of the matrix region is in grey and the capsule is in blue-green. The attached boundary surfaces of the capsule and matrix are highlighted in green and red, respectively. The solid region of the matrix was assigned a mesh with four-node isoperimetric plane strain elements. The free meshing method with mesh control was used such that the mesh was gradually refined approaching the crack and capsule, taking into account the considerably larger region of the matrix in comparison with the capsule. A mesh grid similar to that used in [25] has been introduced considering the present configuration is scaled down with respect to that in [25]. Mesh sensitivity analysis was conducted to ensure the model’s accuracy, balancing computational accuracy and time. As a result, the mesh dimension progressively decreased from 50 µm to 10 µm at the left symmetrical edge, and the meshes on the other three edges (the top, bottom and right) were all 100 µm in size. There were ultimately 51,032 meshes in the matrix overall. The uniform four-node isoperimetric plane strain elements were used with 220 circumferential elements and four elements along the reference thickness (i.e., $t_{c}/R_{c}=$ 0.05) due to the radial symmetry. The debonding of the interface between the capsule and matrix was modeled using four-node zero-thickness cohesive elements (CIEs) with the given elastic and fracture properties, which are available in the finite element package ABAQUS Version 6.5 or higher. Figure 3 illustrates the final mesh grid used in this work. It should be noted that only a quarter of the plate can be modeled instead with nodes relaxing method for the crack for simplicity considering the axial symmetry of the configuration. In this way, the crack path has to be prior known and to align with element boundaries, which are not applicable for the case of arbitrary cracking due to mostly, heterogeneity of matrices, randomly distributed capsules, etc. The XFEM method does not require the mesh to match the geometry of the discontinuities and therefore no considerable mesh refinement is needed in the neighborhood of the crack tip to capture the singular asymptotic fields adequately.

### 2.1. Cohesive Crack Model for Capsule-Matrix Interaction

**Figure 4:**
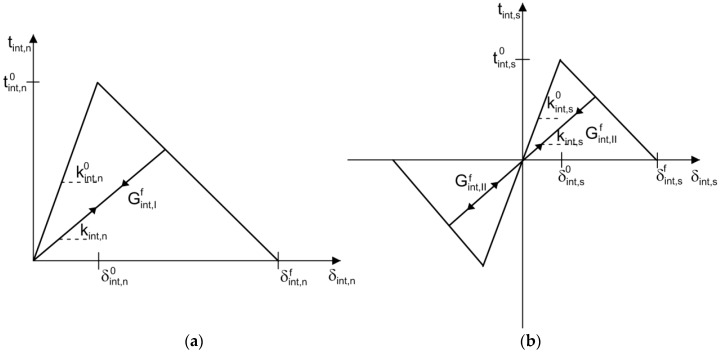
Traction–displacement laws for the interface in the: (**a**) normal direction; and (**b**) tangential direction.

The surface interaction between the capsule and the matrix was modeled using the cohesive crack model [31,32,33], in which energy dissipation occurs in the fracture process zone (FPZ) during fracture. This nonlinear behavior is characterized by a traction–separation law, which is a typical bilinear response, as schematically shown in Figure 4. Here, the subscripts *c*, *m*, and *int* are short for capsule, matrix and interface, respectively. It is assumed that the tractions exist in the normal direction $t_{n}$ and shear direction $t_{s}$ across the crack surface for a 2D case, and the corresponding relative displacements are the crack opening displacement $\delta_{n}$ and the crack sliding displacement $\delta_{s}$. The traction and deformation remain linearly related prior to the onset of damage, at which point the traction reaches a strength of $t_{n}^{0}$ or $t_{s}^{0}$. Then, the traction decreases linearly and monotonically as a function of the corresponding deformation $\delta_{n}$ or $\delta_{s}$. The irreversible degradation of the material is characterized by the progressive reduction in stiffness, defined by the slope $k_{n}$ or $k_{s}$ of the traction–displacement curve, as marked in Figure 4. The covered area is defined as the fracture energy $G_{I}^{f}$ or $G_{\mathrm{II}}^{f}$, which corresponds to *t_n_* = 0 or *t_s_* = 0 and $\delta_{n}=\delta_{n}^{f}$ or $\delta_{s}=\delta_{s}^{f}$.

A scalar index *D*, which is a function of the effective displacement $\delta_{eff}$ (the combined effects of $\delta_{n}$ and $\delta_{s}$), characterizes the overall damage of the crack as given by the following:

(1) $$D_{\mathrm{int}}=\frac{\delta_{\mathrm{int},eff}^{f}\left(\delta_{\mathrm{int},eff}^{\max}-\delta_{\mathrm{int},eff}^{0}\right)}{\delta_{\mathrm{int},eff}^{\max}\left(\delta_{\mathrm{int},eff}^{f}-\delta_{\mathrm{int},eff}^{0}\right)}$$

where $\delta_{\mathrm{int},eff}^{\max}$ is the maximum effective displacement attained during the loading history. $\delta_{\mathrm{int},eff}^{0}$ and $\delta_{\mathrm{int},eff}^{f}$ are the effective displacements at damage initiation and failure corresponding to $\delta_{\mathrm{int},n}^{0}$ and $\delta_{\mathrm{int},s}^{0}$, and $\delta_{\mathrm{int},n}^{f}$ and $\delta_{\mathrm{int},s}^{f}$. The value of *D* varies from 0 to 1, representing an intact and a fully cracked material, respectively.

The effective displacement is:

(2) $$\delta_{\mathrm{int},eff}=\sqrt{{\langle \delta_{\mathrm{int},n}\rangle }^{2}+\delta_{\mathrm{int},s}^{2}}$$

and

(3) $$\langle \delta_{\mathrm{int},n}\rangle =\left\{ \begin{array}{ll} \delta_{\mathrm{int},n}, & \delta_{\mathrm{int},n}\geq 0\\ 0, & \delta_{\mathrm{int},n}<0 \end{array}\right.$$

where 〈⋅〉 is the Macaulay bracket. This definition is based upon the fact that the materials do not undergo damage under pure compression.

Based on the initial stiffness $k_{\mathrm{int},n}^{0}$ and $k_{\mathrm{int},s}^{0}$ of the intact material, the unloading and reloading stiffness $k_{\mathrm{int},n}$ and $k_{\mathrm{int},s}$ can then be determined according to:

(4) $$k_{\mathrm{int},n}=\left(1-D_{\mathrm{int}}\right)k_{\mathrm{int},n}^{0}$$

(5) $$k_{\mathrm{int},s}=\left(1-D_{\mathrm{int}}\right)k_{\mathrm{int},s}^{0}$$

The tractions $t_{\mathrm{int},n}$ and $t_{\mathrm{int},s}$ are written as functions of the corresponding displacements $\delta_{\mathrm{int},n}$ and $\delta_{\mathrm{int},s}$ respectively, as

(6) $$t_{\mathrm{int},n}=\left\{ \begin{array}{ll} \left(1-D_{\mathrm{int}}\right)k_{\mathrm{int},n}^{0}\delta_{\mathrm{int},n} & \delta_{\mathrm{int},n}\geq 0\\ k_{\mathrm{int},n}^{0}\delta_{\mathrm{int},n} & \delta_{\mathrm{int},n}<0 \end{array}\right.$$

(7) $$t_{\mathrm{int},s}=\left(1-D_{\mathrm{int}}\right)k_{\mathrm{int},s}^{0}\delta_{\mathrm{int},s}$$

Concerning the damage initiation, a quadratic nominal stress law is assumed to combine both the effects of normal traction and tangential traction as given by:

(8) $${\left\{\frac{\langle t_{\mathrm{int},n}\rangle }{t_{\mathrm{int},n}^{0}}\right\}}^{2}+{\left\{\frac{t_{\mathrm{int},s}}{t_{\mathrm{int},s}^{0}}\right\}}^{2}=1$$

where $t_{\mathrm{int},n}^{0}$ and $t_{\mathrm{int},s}^{0}$ refer to the maximum value of normal traction and tangential traction of the interface, respectively.

### 2.2. XFEM-Based Cohesive Behavior for Crack Propagation in Matrix 

An extended finite element method was used to model the propagation of a pre-defined crack in a matrix prior to reaching a capsule. This method is based on the concept of partition of unity, which allows local special enriched functions to be incorporated into a finite element approximation to represent discontinuities in a crack [34,35,36]. The enrichment functions typically consist of the near-tip asymptotic functions that representthe singularity around the crack tip and a discontinuous function that describes the abrupt change in displacement across the crack faces [34,35,36]. The approximation for a displacement vector function with the partition of unity enrichment is given by [34,35,36]:

(9) $$u=\sum_{I=1}^{N}N_{I}\left(x\right)\left[u_{I}+H\left(x\right)a_{I}+\sum_{\alpha =1}^{4}F_{\alpha }\left(x\right)b_{I}^{\alpha }\right]$$

where $N_{I}\left(x\right)$ is the usual nodal shape function, $u_{I}$ is the usual nodal displacement vector associated with the continuous part of the finite element solution, $a_{I}$ is the nodal enriched degree of freedom vector, H(x) is the associated discontinuous jump function across the crack surfaces as written as Equation (10) below, $b_{I}^{\alpha }$ is the product of the nodal enriched degree of freedom vector and $F_{\alpha }\left(x\right)$ is the associated elastic asymptotic crack-tip function that can be determined according to Equation (11).

(10) $$H\left(x\right)=\left\{ \begin{array}{ll} 1 & \mathrm{if}\;\left(x-x^{*}\right)\cdot n\geq 0\\ -1 & \mathrm{otherwise} \end{array}\right.$$

where x is a sample (Gauss) point, $x^{*}$ is the point on the crack closest to x, and n is the unit outward normal to the crack at $x^{*}$.

In a polar coordinate system (r,θ) with its origin at the crack tip, $F_{\alpha }\left(x\right)$ is:

(11) $$F_{\alpha }\left(x\right)=\left[\sqrt{r}\sin\frac{\theta }{2},\sqrt{r}\cos\frac{\theta }{2},\sqrt{r}\sin\theta \sin\frac{\theta }{2},\sqrt{r}\sin\theta \cos\frac{\theta }{2}\right]$$

where θ=0 is tangent to the crack at the tip.

**Figure 5:**
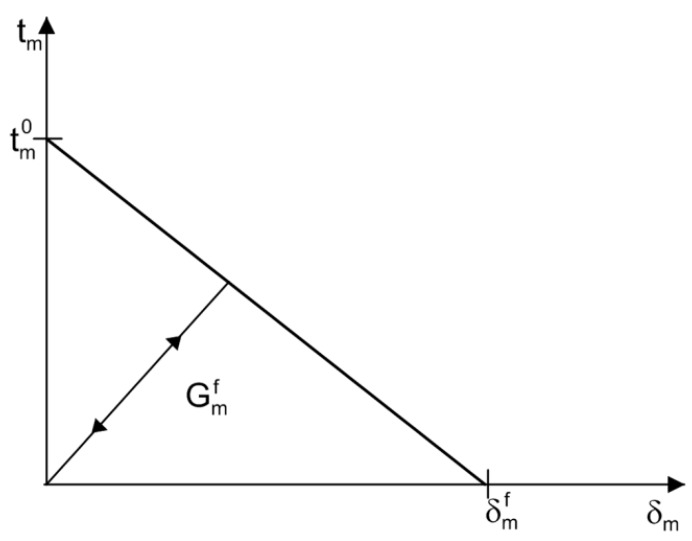
Linear softening law of enriched elements of the matrix.

The damage and failure of an enriched element is based on the cohesive response that consists of a damage initiation criterion and a damage evolution law. A linear elastic response is assumed for the initially undamaged material. Once damage is initiated, the elements will degrade progressively according to a given damage evolution law. Figure 5 schematically depicts a typical and commonly used linear traction–separation response, where the traction $t_{m}=t_{m}^{0}$ starts to decrease to zero corresponding to the maximum displacement $\delta_{m}^{f}$ at which the damage initiation criterion is met. For the sake of simplicity, it is assumed that the enriched elements do not undergo damage under pure compression and that the response is mode-independent.

The maximum principal stress criterion is used for damage initiation in the model, as given by:

(12) $$\left\{\frac{\langle \sigma_{m}^{\max}\rangle }{\sigma_{m}^{0}}\right\}=1$$

Here, $\sigma_{m}^{0}$ and $\sigma_{m}^{\max}$ represent the maximum allowable principal stress and the maximum principal stress at present, respectively.

Similarly, the tractions can be determined by the damage variable $D_{m}$, and the strengths, $t_{m,n}^{0}$ for normal opening and $t_{m,s}^{0}$ for shear sliding of the matrix can be determined:

(13) $$t_{m,n}=\left\{ \begin{array}{ll} \left(1-D_{m}\right)t_{m,n}^{0}, & t_{m,n}^{0}\geq 0\\ t_{m,n}^{0}, & t_{m,n}^{0}<0 \end{array}\right.$$

(14) $$t_{m,s}=\left(1-D_{m}\right)t_{m,s}^{0}$$

$D_{m}$ can be determined in the same way as $D_{\mathrm{int}}$ using Equations (1)–(3).

### 2.3. Input Parameters for Material Properties

The material parameters used in this study are listed in Table 1. Both the effects of elasticity and fracture were examined. The quantity given by the ratio $E_{c}$/$E_{m}$ is hereafter referred to as the elastic ratio to illustrate the influence of the elastic mismatch on cracking and on debonding potential. Various matrix strengths were selected to elucidate crack propagation through the matrix prior to the onset of debonding at an interface. The host strengths correspond to a wide range of materials, including cement-based materials (mean value of 3.5 MPa [37]) and polymer matrices such as epoxy resins (39 ± 4 MPa in [38]). Various interface strengths were modeled, ranging from weak bonding (0.1 MPa) to perfect bonding (10 times of matrix strength, i.e., 35 MPa) between the capsule and matrix. Regarding the fracture properties of matrix, the literatures provide tentative values of $G_{m}^{f}$ for normal concrete (0.15 N/mm in [37]) and for cement-based materials with a certain additives, such as the well-known fiber-reinforced cement (2.1 N/mm in [39]). 

**Figure 6:**
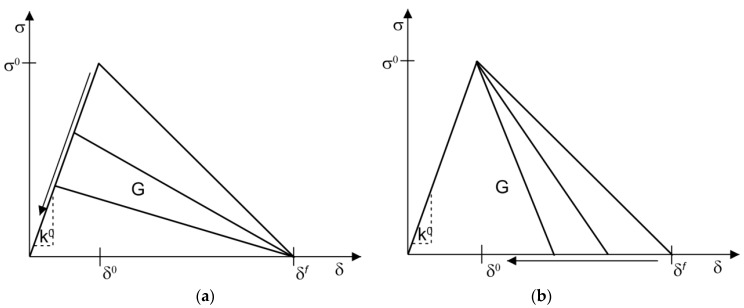
Modifications of the cohesive law for both the matrix and the interface: (**a**) reduction of the maximum strength $\sigma^{0}$ with $\delta^{f}=\mathrm{constant}$; and (**b**) reduction of the maximum displacement $\delta^{f}$ with $\sigma^{0}=\mathrm{constant}$.

Taking into account $G_{m}^{f}=\frac{1}{2}t_{m}^{0}\delta_{m}^{f}$ (or $G_{\mathrm{int}}^{f}=\frac{1}{2}t_{\mathrm{int}}^{0}\delta_{\mathrm{int}}^{f}$), the modification of $G_{m}^{f}$ (or $G_{\mathrm{int}}^{f}$) was performed by changing either: (a) the strength $t_{m}^{0}$ (or $t_{\mathrm{int}}^{0}$), while $\delta_{m}^{f}\left(\mathrm{or}\;\delta_{\mathrm{int}}^{f}\right)=\mathrm{constant}$; or (b) the maximum displacement $\delta_{m}^{f}\left(\mathrm{or}\;\delta_{\mathrm{int}}^{f}\right)$, while $t_{m}^{0}$ (or $t_{\mathrm{int}}^{0}=$ constant), as presented in Figure 6. For both the matrix and capsule, the shear fracture properties were simply assumed to be the same as the normal fracture properties: $t_{m,n}^{0}=t_{m,s}^{0}$ (or $t_{\mathrm{int},n}^{0}=t_{\mathrm{int},s}^{0}$), $G_{m,n}^{f}=G_{m,s}^{f}$ (or $G_{\mathrm{int},n}^{f}=G_{\mathrm{int},s}^{f}$) and $k_{m,n}^{0}=k_{m,s}^{0}$ (or $k_{\mathrm{int},n}^{0}=k_{\mathrm{int},s}^{0}$) for mode-independent response. Unless stated otherwise, the reference example values were ∑= 3 MPa, $t_{c}/R_{c}=$ 0.05, $l_{0}=$ 325 µm, *s* = 350 µm, $t_{m}^{0}=$ 3.5 MPa, $k_{m,n}^{0}=k_{m,s}^{0}=$ 25,000 MPa/mm, $E_{c}/E_{m}=$ 1, $G_{m}^{0}=G_{\mathrm{int}}^{0}=$ 0.15 N/mm, and $t_{\mathrm{int}}^{0}/t_{m}^{0}=$ 10 for perfect bonding or $t_{\mathrm{int}}^{0}/t_{m}^{0}=$ 1 for imperfect bonding, respectively. The Poisson’s ratio ν was kept at 0.18 throughout.

## 3. Results and Discussion

### 3.1. Crack Extension in Matrix for Perfect Bonding

**Figure 7:**
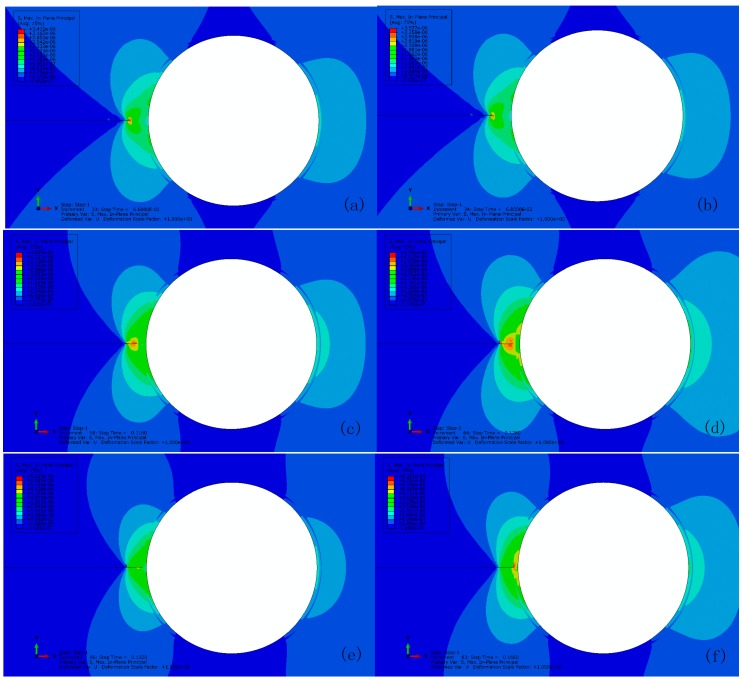
Crack extension for the reference with perfect bonding at: (**a**) 0.066; (**b**) 0.068; (**c**) 0.116; (**d**) 0.128; (**e**) 0.132; and (**f**) 0.166 of σ/∑.

Figure 7 shows the typical maximum in-plane stress principle in the vicinity of crack tip at interval of progressive crack extension until it reaches to the capsule wall for the reference with perfect bonding (i.e., $t_{\mathrm{int}}^{0}/t_{m}^{0}=$10). To track every step of crack extension, the output was recorded at time interval of 0.002 of σ/∑. The stress concentration is observed at the crack tip (see Figure 7a) prior to its propagation when the maximum stress increases to 3.5 MPa (see Figure 7b). The crack has to propagate across an entire element at a time to avoid the need to model the stress singularity. Unloading occurs to the stress concentrated elements when the crack propagates and the stress concentration shifts to the new crack tip, e.g., crack extension as shown in Figure 7c,d. Depending on the respective mechanical properties, the crack will either propagate along the interface or rupture the wall when the crack reaches to the capsule wall (see Figure 7e). Here, the crack cannot propagate in either way due to a quite high interface strength (i.e., $t_{\mathrm{int}}^{0}/t_{m}^{0}=$ 10) and the elastic response of capsule wall as assumed. Therefore, the stress accumulates between the interface and the capsule wall. In other words, the crack can propagate through the wall if its strength is less than 6.3 MPa as shown in Figure 7f once the interface is sufficiently strong to avoid debonding to occur prior to the wall breakage.

**Figure 8:**
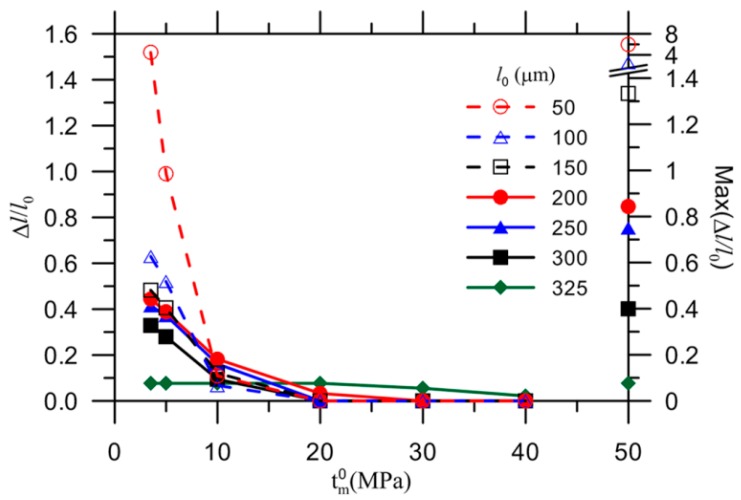
Ultimate crack length ratio at interval of matrix strength with respect to its maximum crack length ratio.

Figure 8 shows the normalized ultimate crack extension with respect to its initial length, denoted by $\Delta l/l_{0}=\left(l-l_{0}\right)/l_{0}$ and labeled on the left y-axis, for various initial crack sizes, from 50 µm up to 325 µm, as a function of the matrix strength. The maximum crack growth up to the capsule wall (i.e., *l* = 350 µm) is presented on the right y-axis. The value of $\Delta l/l_{0}$ initially exhibits a sharp decrease with increasing matrix strength for $t_{m}^{0}\leq$ 10 MPa. The rate of decrease eventually slows down, with the exception of the crack 325 µm in length, which remains at its maximum value (i.e., 0.077) until the matrix strength increases to 20 MPa. For the remaining crack sizes, $\Delta l/l_{0}$ is less than its maximum value regardless of the matrix strength. These results imply that the initial crack length should be no less than 325 µm and the matrix strength should be no higher than 20 MPa for the crack to propagate to the capsule at the present load and boundary conditions. Thus, the initial crack length was fixed at 325 µm in the subsequent study to keep the focus on crack propagation through the matrix, resulting in either capsule rupture or capsule bypass.

**Figure 9:**
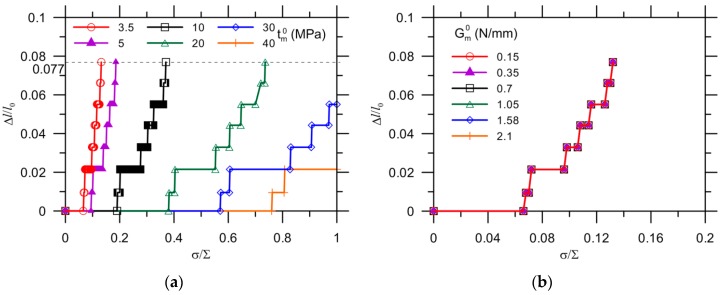
Effect of fracture properties on crack evolution by modification of: (**a**) strength; and (**b**) maximum displacement of the matrix.

Figure 9a shows the detailed crack propagation process, as normalized by its initial length, *l*_0_ = 325 i.e., µm, for a load applied to matrices with various strengths. The crack extended to the capsule wall for cases in which $\Delta l/l_{0}$ = 0.077 (0.055 or 0.022) provided the matrix strength was no more than 20 MPa, 30 MPa, and 40 MPa, respectively. A higher load increase was necessary to initiate crack propagation for an increase of matrix strength.

The influence of the fracture energy of the matrix with constant strength $t_{m}^{0}$ = 3.5 MPa on crack extension is presented in Figure 9b. The fracture energy ranged from values representing normal, plain cement [37] to values for fiber-reinforced cement to ensure universal coverage of materials [39]. However, the fracture energies selected did not show an obvious influence on the crack propagation. This can be explained by the choice of the criteria for crack initiation, which is stress controlled in this model, as given by Equation (12), while the fracture energy strongly affects the damage evolution in course of displacement, as characterized by the damage index defined in Equation (1). Besides, the crack extension is much smaller than its full length, i.e., 25/(325 + 25) = 0.071. Likewise, the effect of fracture energy on a capsule with constant strength on the likelihood of debonding is insignificant until 0.2–0.4 of the relative crack extension is reached with respect to the circumference of the full interface [25].

**Figure 10:**
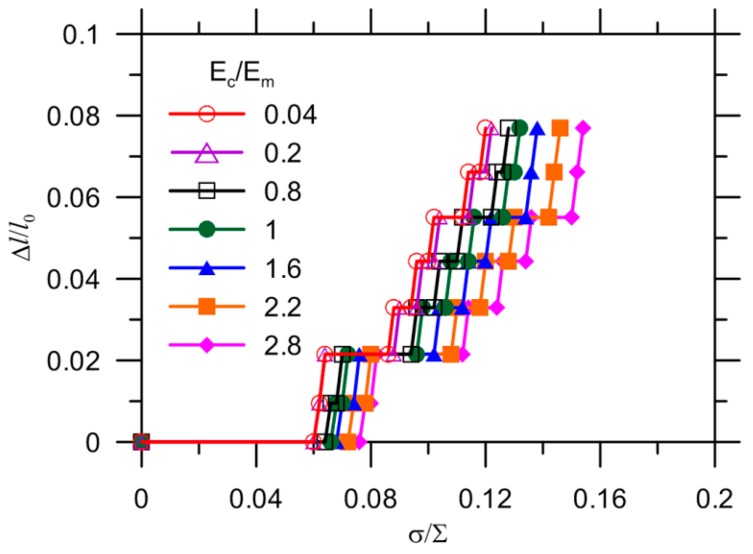
Effect of Young’s modulus ratio on crack extension.

The effect of the Young’s modulus ratio $E_{c}/E_{m}$ on the crack extension in the matrix is shown in Figure 10. As discussed in [25], an appropriate range of values for the capsule elasticity were used, ranging from polymer elasticity (≥1 GPa) [40] to the elasticity of stiffer materials, such as ceramic or conventional glass (≤70 GPa) [41]. The Young’s modulus was kept at 25 GPa for the matrix material as a reference. The increase of $E_{c}/E_{m}$ delays the crack initiation and growth but does not change the ultimate crack length. In other words, the crack was always able to propagate to the capsule with the Young’s modulus selected in the present study. Compared with the aforementioned results, it is proposed that crack extension in a matrix is influenced significantly by the strength of the matrix, which governs the ultimate crack length, whereas the Young’s modulus ratio $E_{c}/E_{m}$ only affects the rate of crack propagation. It should be noted that the presence of defects (inclusions or voids) strongly affects the fracture mechanism because of the interaction between the defects and the cracks. A crack is either attracted or repelled by inclusions or voids depending on the comparative rigidity of the defects to the matrix. The final crack trajectory is a result of the combined effect of the inclusions or voids. In the present model, the void left by the capsule attracts the crack but the crack can be either attracted or deflected depending on the stiffness of the capsule wall.

**Figure 11:**
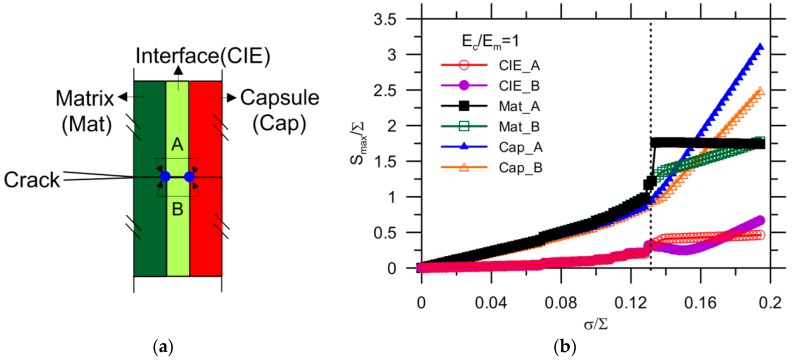
(**a**) Spatial location of targeted nodes; and (**b**) maximum nodal stress in-plane for the interface, matrix and capsule.

The nodal stress values of the interface, matrix and capsule, labeled “_A” and “_B” for the upper and lower elements, respectively, as shown in Figure 11a, are collected and presented in Figure 11b. The other elastic and fracture indexes were all kept the same as reference values. The stress progressively increases with applied load, and the interface exhibits a quite low stress compared to that of the matrix and capsule. One striking feature is that, for the same phase, the nodal stress curves _A and _B are coincident for the elements until a certain load, i.e., σ/∑ = 0.132, as marked by a dashed line in Figure 11b, after which the stress curves diverge. The stress of the matrix and capsule are very close until 0.132 of σ/∑, while the interface stays at a comparatively low stress. This demonstrates that the capsule and matrix are attached quite well, as there is a complete stress transition between the two. The reason for the abrupt change of stress at σ/∑ = 0.132 is that the crack tip reaches the boundary of the matrix but cannot propagate through the CIEs of interface using XFEM and hereafter the crack is characterized by the damage of CIEs. The stress at which the curves split are taken as the critical stress $S_{cr}$, corresponding to the crack reaching the interface. 

**Figure 12:**
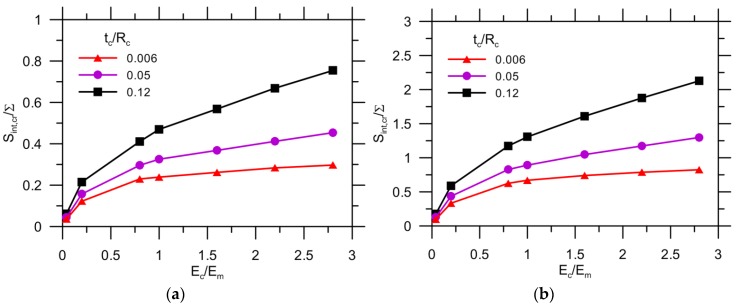
Critical stress of interface for: (**a**) $t_{m}^{0}=$ 3.5 MPa; and (**b**) $t_{m}^{0}=$ 10 MPa.

Figure 12 shows the effect of the Young’s modulus ratio on the critical stress of the interface, which is normalized by the remote load applied to a matrix with strength of either 3.5 MPa (Figure 12a) or 10 MPa (Figure 12b). The higher thickness of the capsule wall results in a higher critical stress, which increases with $E_{c}/E_{m}$ because of concentrated stress. The effect of $E_{c}/E_{m}$ on the critical stress becomes more significant with an increase of $t_{c}/R_{c}$. Similarly, the curves for the matrix with $t_{m}^{0}$ = 10 MPa exhibit the same trend, but the critical stress increases by approximately three times when $t_{m}^{0}$ = 3.5 MPa. Thus, the critical stress of the interface $S_{\mathrm{int},cr}$, as one of the crucial requirements for preventing detachment of the matrix from the capsule, can be determined for materials design. The other requirement is the critical stress of the capsule $S_{c,cr}$ because the onset of debonding depends on the comparative strength between the capsule and interface ($S_{c}/S_{\mathrm{int}}$), which will be discussed further. For instance, given $t_{m}^{0}$ = 3.5 MPa, $E_{c}/E_{m}$ = 1 and $t_{c}/R_{c}$ = 0.05, the critical strength $S_{\mathrm{int},cr}$ is 0.33 ×∑.

**Figure 13:**
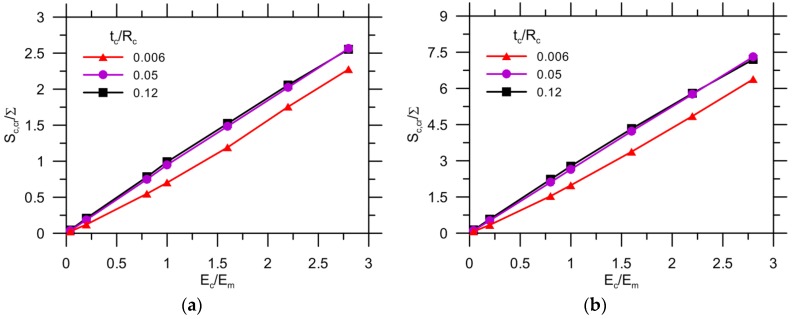
Critical stress of capsule for: (**a**) $t_{m}^{0}=$ 3.5 MPa; and (**b**) $t_{m}^{0}=$ 10 MPa.

Figure 13 depicts the result of the critical stress of the capsule for both $t_{m}^{0}$ = 3.5 MPa (Figure 13a) and $t_{m}^{0}$ = 10 MPa (Figure 13b). The value of $S_{cr}/\sum$ increases almost linearly with the elastic ratio for the three levels of thickness: a value of 0.006 for $t_{c}/R_{c}$ exhibits quite a low $S_{cr}/\sum$ ratio for the same $E_{c}/E_{m}$ value when compared with other results. However, the stress curves are nearly coincident with a further increase of $t_{c}/R_{c}$ from 0.05 to 0.12. For $t_{m}^{0}$ = 10 MPa, the curve exhibits a same trend, but the critical stress is approximately three times higher than with $t_{m}^{0}$ = 3.5 MPa. It can be concluded that there is an optimal selection for the capsule thickness, i.e., 0.05 of $t_{c}/R_{c}$, for optimal cost saving. Other than this, the critical capsule stress $S_{c,cr}$ is known to ensure that the capsule can be ruptured by crack propagation, provided the interface is strong enough. For instance, the critical stress is less than one times the applied load ($S_{c,cr}/\sum <$ 1) provided $t_{m}^{0}$ = 3.5 MPa, $E_{c}/E_{m}=1$ and $t_{c}/R_{c}$ = 0.05.

In general, the potential for capsule breakage is dependent on the comparative strength between the capsule and interface, provided a crack can reach the capsule with a given load, as discussed above. This can be written as [42,43,44,45]:

(15) $$S_{c}/S_{\mathrm{int}}\leq S_{cr,c}/S_{cr,\mathrm{int}}$$

where $S_{c}$ and $S_{\mathrm{int}}$ are the design strengths of the capsule and interface, respectively. Likewise, $S_{cr,c}$ and $S_{cr,\mathrm{int}}$ are the critical stresses obtained in the model for the capsule and interface, respectively. Taking the reference as an example, $S_{c}/S_{\mathrm{int}}$ should be no more than 1/0.33 ≈ 3 inaccordance with Figure 12a and Figure 13a.

### 3.2. Debonding Due to Capsule-Matrix Interaction

**Figure 14:**
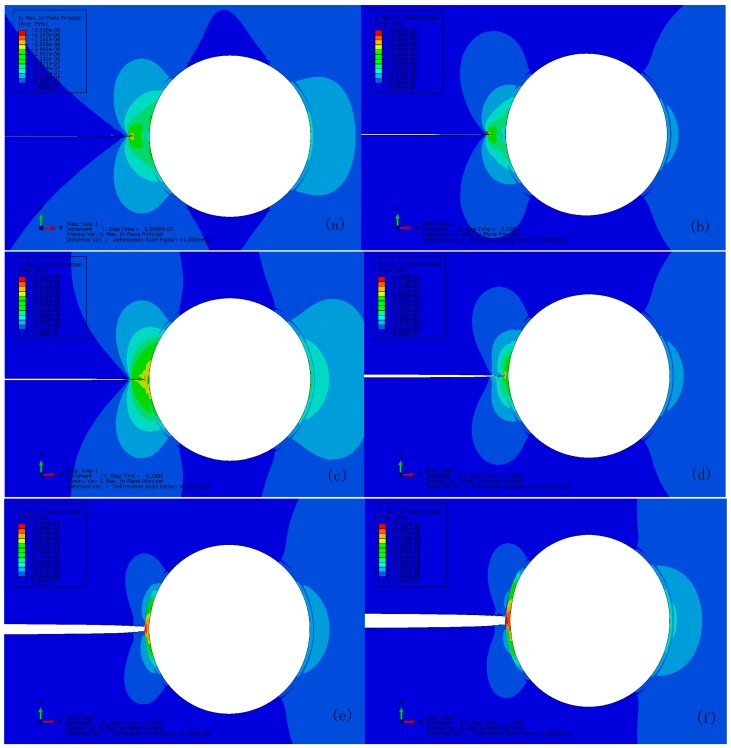
Crack extension for the reference with imperfect bonding ($t_{\mathrm{int}}^{0}/t_{m}^{0}$ = 1) at: (**a**) 0.05; (**b**) 0.1; (**c**) 0.15; (**d**) 0.25; (**e**) 0.75; and (**f**) 1.0 of σ/∑.

**Figure 15:**
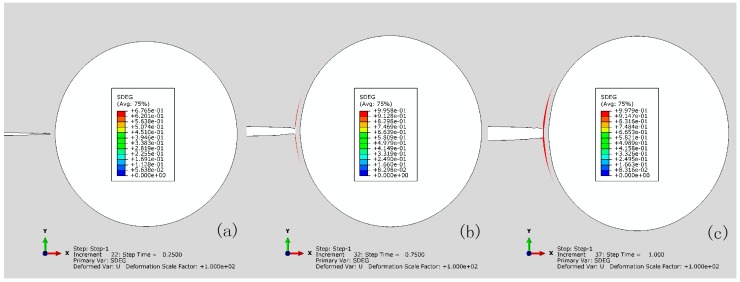
SDEG of CIEs for the reference with imperfect bonding ($t_{\mathrm{int}}^{0}/t_{m}^{0}$ = 1) at: (**a**) 0.25; (**b**) 0.75; and (**c**) 1.0 of σ/∑.

Figure 14 shows the progressive crack extension for the reference with imperfect bonding (i.e., $t_{\mathrm{int}}^{0}/t_{m}^{0}$ = 1). Stress concentration is observed in the capsule wall as the crack extends along the interface and the debonding initiates at 0.25 of σ/∑, as characterized by damage of CIEs, i.e., the scalar damage variable (SDEG) higher than zero, as shown in Figure 15, comparing to the condition of perfect bonding (see Figure 7) where no damage of CIEs occurs. 

**Figure 16:**
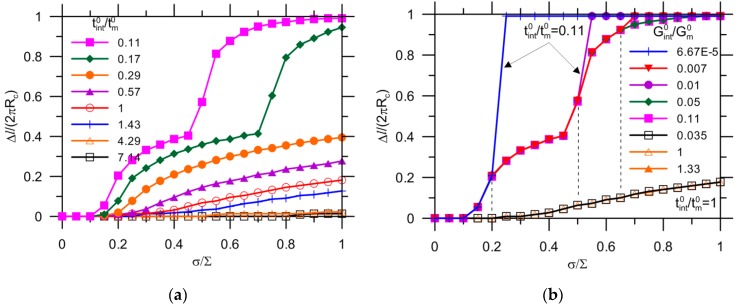
Effect of fracture properties on debonding by modification of: (**a**) strength; and (**b**) maximum displacement of interface.

**Figure 17:**
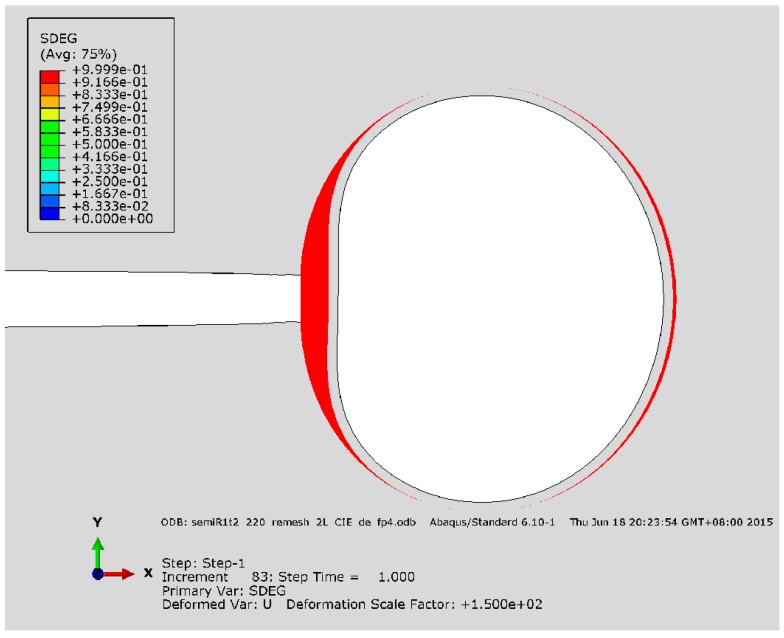
SDEG ofcohesive elements at σ/∑=1.

Figure 16 shows the debonding evolution as a function of the applied load for the reference with a variety of fracture properties of the interface. The effect of the strength $t_{\mathrm{int}}^{0}$ with $\delta_{\mathrm{int}}^{0}$ kept constant on the crack growth has been presented in Figure 16a. Conversely, the results for a variety of fracture energies $G_{\mathrm{int}}^{0}$ with $t_{\mathrm{int}}^{0}$ kept constant are reported in Figure 16b. The onset of the capsule debonding shifts to a higher load for a higher interfacial strength. The curves can be divided into two groups by 0.29 of $t_{\mathrm{int}}^{0}/t_{m}^{0}$. This agrees well with the results for a stress of $S_{cr}=$ 0.33⋅∑= 0.33 × 3 ≈ 1.0 ($t_{\mathrm{int}}^{0}$ = 0.29∙$t_{m}^{0}$ = 0.29 × 3.5 ≈ 1.0) as shown in Figure 12a. For the condition $t_{\mathrm{int}}^{0}/t_{m}^{0}<$ 0.29, the crack extension breaks into two stages, with an abrupt growth at 0.45 and 0.7 of σ/∑ for $t_{\mathrm{int}}^{0}/t_{m}^{0}$ = 0.11 and 0.17, respectively. This can be explained by debonding also initiating from other locations due to stress concentration, as shown in Figure 17. The cracks ultimately merge together, leading to complete detachment of the capsule–matrix, i.e., Δl/(2$\pi R_{c}$) = 1. Regarding the $t_{\mathrm{int}}^{0}/t_{m}^{0}\geq$ 0.29 condition, debonding still occurs because capsule response is assumed to be elastic in the present model. When $t_{\mathrm{int}}^{0}$ increases to 7.14 times that of the matrix strength, the capsule–matrix debonding does not initiate regardless of the capsule mechanical properties under given load conditions at present.

A wide range of fracture energies were analyzed for two strength intervals of the interface, as depicted in Figure 16b. The onset and extension of debonding do not depend on the value of fracture energy for $t_{\mathrm{int}}^{0}/t_{m}^{0}=$ 1. The crack propagation ends with Δl/(2$\pi R_{c}$) = 0.2, which might be too small to differentiate the influence of $G_{\mathrm{int}}^{0}/G_{m}^{0}$. It was reported that debonding collapses to the same curves before that 0.2–0.4 of the circumference of the interface is detached [25]. In other words, there seems to exist a limit value of the toughness of bonding after which the crack growth along the interface is hardly affected [25]. A smaller value of $t_{\mathrm{int}}^{0}/t_{m}^{0}$ = 0.11 was selected for further analysis, and debonding initiated at a lower load than expected. It exhibits separate trend of crack extension after Δl/(2$\pi R_{c}$) = 0.2. The critical stage shifts further to a load as high as 0.5 followed by 0.65 of σ/∑, as highlighted in dashed lines in Figure 16b. However, the onset of debonding does not change with fracture energy for all cases. 

**Figure 18:**
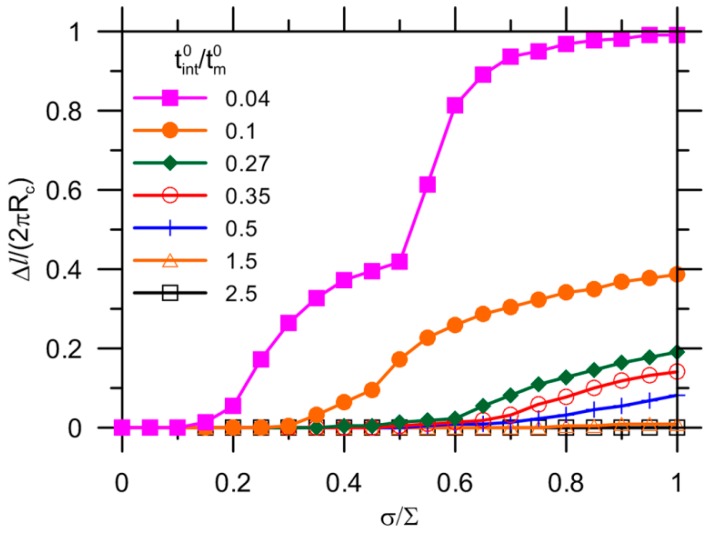
Effect of interface strength on debonding evolution for $t_{m}^{0}=10$ MPa.

Figure 18 shows the debonding evolution for the reference but with $t_{m}^{0}=$ 10 MPa. Again, the higher strength results in the initiation of debonding at the higher applied load. The critical condition for debonding is $t_{\mathrm{int}}^{0}/t_{m}^{0}=$ 0.27 according to $S_{cr}/\sum =$ 0.89, as shown in Figure 12b. Likewise, debonding occurs at multiple locations for $t_{\mathrm{int}}^{0}/t_{m}^{0}>$ 0.27 and otherwise. The detachment between the capsule and the matrix can be perfectly avoided when the value of $t_{\mathrm{int}}^{0}/t_{m}^{0}$ increases to 1.5. A quite low strength of the interface, i.e., $t_{\mathrm{int}}^{0}/t_{m}^{0}=$ 0.04, still leads to complete debonding.

**Figure 19:**
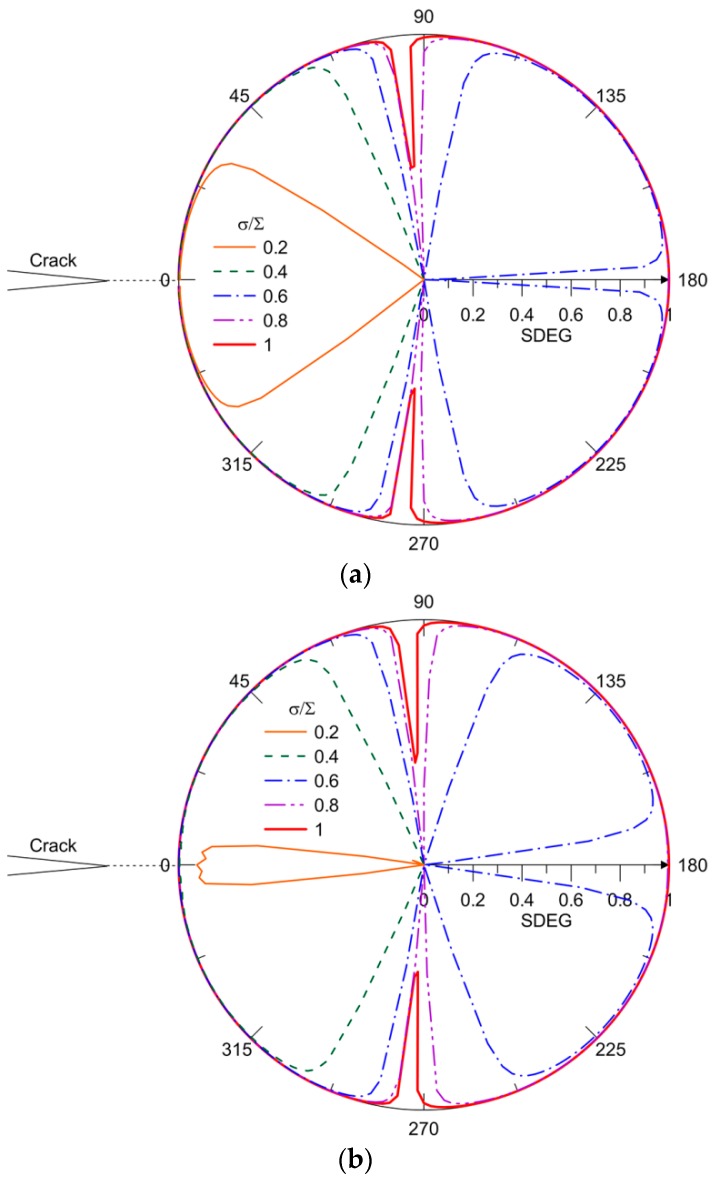
SDEG distribution on capsule at load intervals for: (**a**) $t_{\mathrm{int}}^{0}/t_{m}^{0}=$ 0.11 and $t_{m}^{0}=$ 3.5; and (**b**) $t_{\mathrm{int}}^{0}/t_{m}^{0}=$ 0.04 and $t_{m}^{0}=$ 10.

Figure 19 shows that the SDEG spatially distributes along the capsule–matrix interface at load intervals corresponding to the first complete debonding, as shown in Figure 16a (i.e., $t_{\mathrm{int}}^{0}/t_{m}^{0}=$ 0.11 and $t_{m}^{0}=$ 3.5) and Figure 18 (i.e., $t_{\mathrm{int}}^{0}/t_{m}^{0}=$ 0.04 and $t_{m}^{0}=$ 10). The damage of elements at the interface initiates when the crack tip approaches, i.e., θ= 0°. The detachment propagates progressively along the interface with the applied load until it is close to the compression stressed zone, where the direction of the cross section is nearly parallel to the remote tensile load, i.e., θ= 90° and 270°. At that point, the debonding initiates from the other side by σ/∑= 0.6, as highlighted in the blue dashed line in Figure 19a. The exception is at the vicinity of θ= 180°, which remains intact due to the resultant stress state. With further load increases, the dominating stress in the vicinity of θ= 180° transitions to tension and, thus, debonding occurs there. For the compression stressed zone, the intact section narrows with a load increase. The remaining CIEs also initiate damage with a minimum SDEG = 0.5 at σ/∑= 1, and at this point, complete debonding occurs. The same trend is observed in Figure 19b, but one difference is that the SDEG of CIEs is approximately 0.8–0.9 at σ/∑= 0.2 rather than 1 for $t_{\mathrm{int}}^{0}/t_{m}^{0}=$ 0.11 and $t_{m}^{0}=$ 3.5, as shown in Figure 19a. The debonding propagates in the same way for various load intervals until the SDEG increases to 1. 

**Figure 20:**
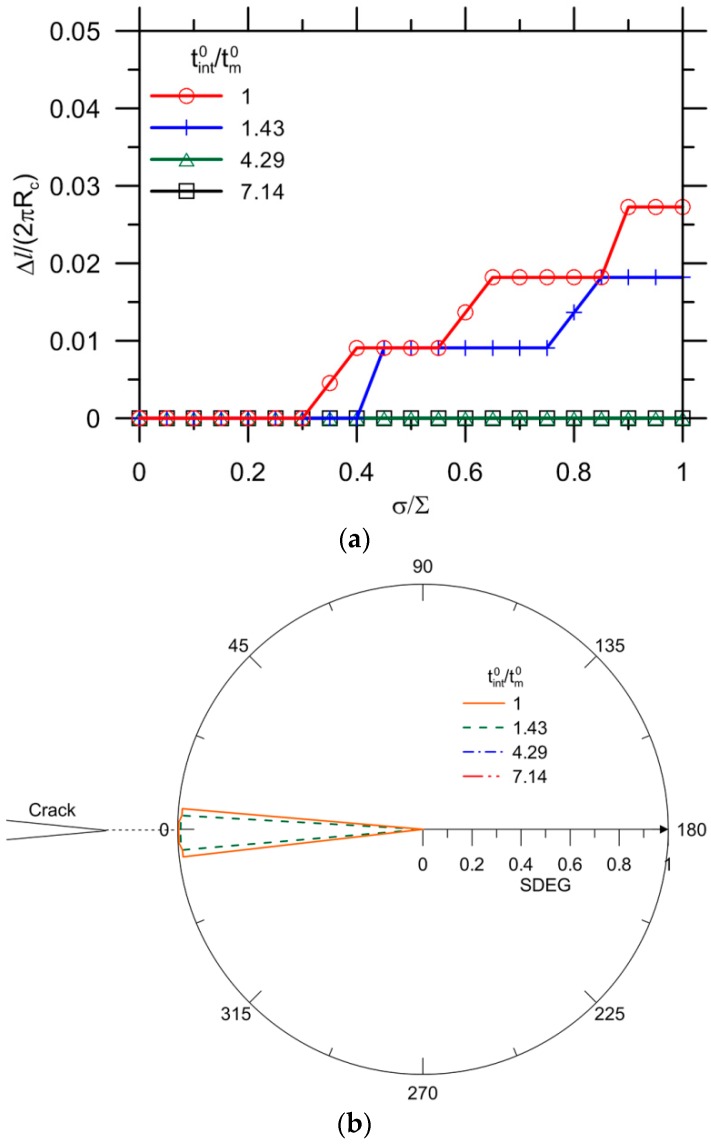
(**a**) Debonding extension for $E_{c}/E_{m}=$ 0.4; and (**b**) corresponding SDEG at σ/∑= 1.

Figure 20a shows the results for $E_{c}/E_{m}=$ 0.4, while the other indexes are all kept constant as reference values. A decrease of the Young’s modulus ratio substantially delays the onset and extension of debonding. No complete debonding is observed for the selected strength ratios of $t_{\mathrm{int}}^{0}/t_{m}^{0}$. The capsule–matrix detachment can be prevented when $t_{\mathrm{int}}^{0}/t_{m}^{0}$ increases to 4.29. 

Figure 20b shows the final distribution of SDEG values along the interface for various $t_{\mathrm{int}}^{0}/t_{m}^{0}$ ratios corresponding to Figure 20a. Only partial debonding is observed at the vicinity of θ= 0° for $t_{\mathrm{int}}^{0}/t_{m}^{0}=$ 1 and 1.43, beyond which the onset of debonding does not occur.

**Figure 21:**
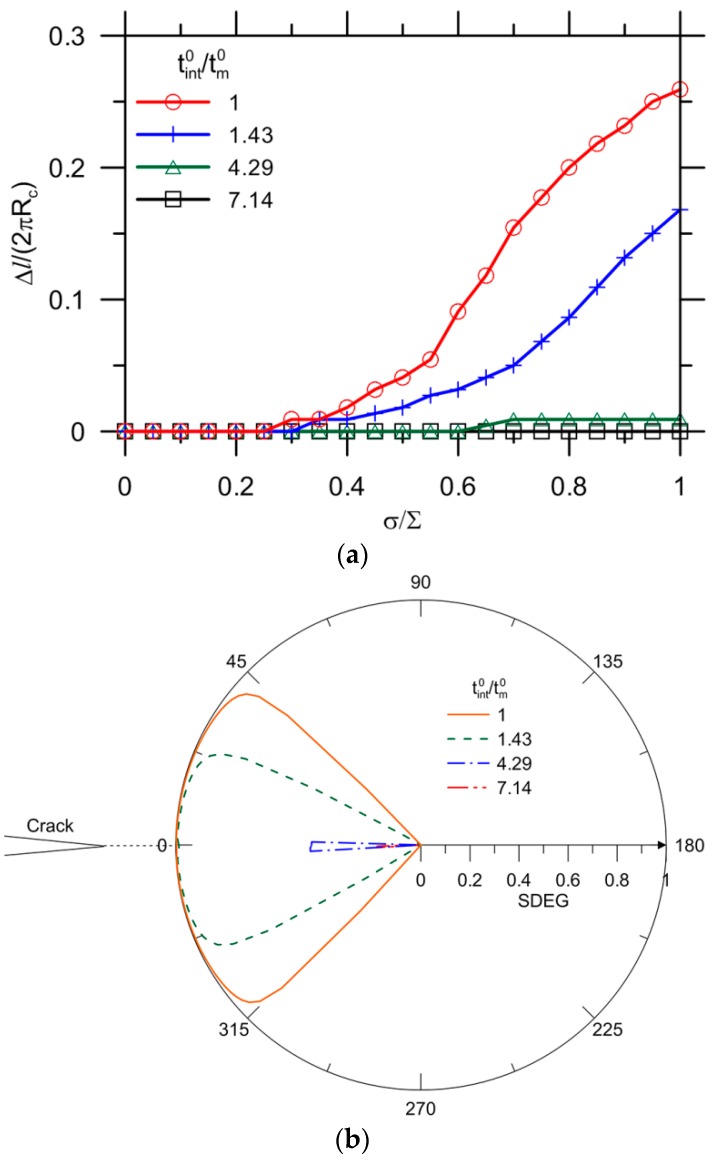
(**a**) Debonding extension for $t_{c}/R_{c}=$ 0.12; and (**b**) corresponding SDEG at σ/∑= 1.

For the capsule with a higher thickness, i.e., $t_{c}/R_{c}=$ 0.12, similar results are observed, as shown in Figure 21a. No debonding is observed for an interface with strength higher than 7.14 times the strength of the matrix. Given $t_{\mathrm{int}}^{0}/t_{m}^{0}=$ 1, the ultimate crack growth Δl/(2$\pi R_{c}$) = 0.26 is greater than the reference value of 0.19 (i.e., $t_{m}^{0}=3.5$ MPa), followed by a value of 0.15 for higher matrix strength (i.e., $t_{m}^{0}=10$ MPa) and of 0.03 for a decrease of Young’s modulus ratio (i.e.,$E_{c}/E_{m}=0.4$). In Figure 21b, the debonding occurs in the vicinity of θ= 0° for $t_{\mathrm{int}}^{0}/t_{m}^{0}=$ 1, 1.43 and 4.29, beyond which the onset of debonding is avoided.

## 4. Conclusions

A mesomechanical model was proposed in this study to simulate the fracture behavior of encapsulation-based healing materials. The crack propagation in a matrix and the capsule–matrix interaction were investigated numerically using the extended finite element method (XFEM) combined with a cohesive zone model (CZM) in a 2D configuration consisting of an infinite matrix with an embedded crack and a capsule nearby. A remote uniaxial tensile and uniform load was applied on the system, and a typical bilinear cohesive law was used in the model. The effect of geometry, elastic parameters and fracture properties on crack extension in the matrix and the potential of debonding were investigated in the parametric study. The following conclusions can be drawn from the analysis: 

(i) For crack propagation in matrix prior to reaching a capsule:

To ensure that the crack can propagate and reach a capsule, the initial crack length should be no less than 325 µm, and the matrix strength should be no higher than 20 MPa for the present loading conditions.

Regarding the fracture energy of matrix, the modification of its strength $t_{m}^{0}$ ($\delta_{m}^{f}=$ constant) exhibits a more significant effect on the ultimate crack length than changing the maximum displacement $\delta_{m}^{f}$ ($t_{m}^{0}=$ constant), which is because a stress-based criterion for crack initiation was used in this model.

The Young’s modulus ratio $E_{c}/E_{m}$ only affects the rate of crack propagation and does not change the ultimate crack length. 

(ii) For the potential of debonding due to capsule–matrix interaction:

The stress of the interface and capsule as the crack reaches the capsule can be known and used as the critical stress for material design. 

$t_{c}/R_{c}=$ 0.05 is an optimal selection for the capsule thickness value according to the critical stress field.

The potential for capsule breakage or debonding is dependent on the comparative strength of the capsule and interface ($S_{c}/S_{\mathrm{int}}$), provided a crack can reach the capsule with a given load. The critical value of $S_{cr,c}/S_{cr,\mathrm{int}}$ for capsule breakage can be obtained from the model for design as $S_{c}/S_{\mathrm{int}}\leq S_{cr,c}/S_{cr,\mathrm{int}}$, whereas debonding occurs otherwise.

For the reference example, debonding can be avoided provided $t_{\mathrm{int}}^{0}/t_{m}^{0}\geq$ 7.14, regardless of the capsule’s mechanical properties for the present load and boundary conditions.

Some limitations of the present results are the following:(i) no arbitrary cracking due to, e.g., heterogeneous and anisotropic matrix, multiple capsules with a random distribution, etc., has been included; (ii) only mode-I is assumed to be the dominant failure mode; (iii) the prescribed load is uniform and uniaxial tension; (iv) the influence of Poisson’s ratio is not considered; (v) the fracture indexes are kept the same in the normal and tangential direction; and (vi) the capsule response is assumed to be linear elastic. These factors can be included in the present model framework for more general considerations and the associated study is in process. 

## Figures and Tables

**Table 1:** Input parameters used in the analysis.

Solid	Young’s Modulus (GPa)	Strength (MPa)	Fracture Energy (N/mm)
Matrix	$E_{m}$ = 25	$t_{m}^{0}$ = 3.5, 5, 10, 20, 30, 40	$\begin{array}{l} \mathrm{for}\;\delta_{m}^{f}=\;\mathrm{constant}:\\ G_{m}^{f}=0.15,0.21,0.43,0.86,1.29,1.71\\ \mathrm{for}\;t_{m}^{0}=\;\mathrm{constant}:\\ G_{m}^{f}=0.15,0.35,0.7,1.05,1.58,2.10 \end{array}$
capsule	$\begin{array}{l} E_{c}=1,5,20,25,\\ 40,55,70\\ (E_{c}/E_{m}=0.04,\\ 0.2,0.8,1,1.6,\\ 2.2,2.8) \end{array}$	-	-
Interface	$E_{\mathrm{int}}$ = 25	$\begin{array}{l} \mathrm{for}\;t_{m}^{0}=3.5:\\ t_{\mathrm{int}}^{0}=0.4,0.6,1,2,3.5,\\ 5,15,25\\ (t_{\mathrm{int}}^{0}/t_{m}^{0}=0.11,0.17,\\ 0.29,0.57,1,1.43,\\ 4.29,7.14)\\ \mathrm{for}t_{m}^{0}=10:\\ t_{\mathrm{int}}^{0}=0.4,1,2.7,3.5,\\ 5,15,25\\ (t_{\mathrm{int}}^{0}/t_{m}^{0}=0.04,0.1,0.27,\\ 0.35,0.5,1.5,2.5,\\ 4.29,7.14) \end{array}$	$\begin{array}{l} \mathrm{for}\;\delta_{\mathrm{int}}^{f}=\;\mathrm{constant}:\\ G_{\mathrm{int}}^{f}=0.0171,0.0257,0.0429,0.0857,\\ 0.15,0.214,0.643,1.071)\\ (G_{\mathrm{int}}^{f}/G_{m}^{f}=0.11,0.17,0.29,0.57,1,\\ 1.43,4.29,7.14) \end{array}$ $\begin{array}{l} \mathrm{for}t_{\mathrm{int}}^{0}=\;\mathrm{constant}\;(\;t_{\mathrm{int}}^{0}/t_{m}^{0}=1):\\ G_{\mathrm{int}}^{f}=0.00525,0.15,0.20\\ \left(G_{\mathrm{int}}^{f}/G_{m}^{f}=0.035,1,1.33\right)\\ \mathrm{for}t_{\mathrm{int}}^{0}=\;\mathrm{constant}\;(\;t_{\mathrm{int}}^{0}/t_{m}^{0}=0.11):\\ G_{\mathrm{int}}^{f}=1e-5,0.001,0.002,0.008,0.171\\ (G_{\mathrm{int}}^{f}/G_{m}^{f}=6.67e-5,0.007,0.013,\\ 0.053,0.114) \end{array}$
